# H-FABP Levels and Psycho-Emotional Improvement of CABG Patients during Cardiac Rehabilitation

**DOI:** 10.3390/jcdd9080242

**Published:** 2022-07-28

**Authors:** Razan Al Namat, Dina Al Namat, Manuela Ciocoiu, Marius Valeriu Hînganu, Laurențiu Șorodoc, Victorița Șorodoc, Liliana Georgeta Foia, Laura Florea, Cristiana Vlad, Ana Tănasă, Mihai Constantin, Daniel Cioloca, Minerva Codruța Bădescu, Amin Bazyani, Maura Felea

**Affiliations:** 1 Faculty of Medicine, “Grigore T. Popa” University of Medicine and Pharmacy Iași, 700115 Iasi, Romania; dina.alnamat@gmail.com (D.A.N.); manuela.ciocoiu@umfiasi.ro (M.C.); marius.hinganu@umfiasi.ro (M.V.H.); laurentiu.sorodoc@umfiasi.ro (L.Ș.); victorita.sorodoc@umfiasi.ro (V.Ș.); lilifoia@yahoo.com (L.G.F.); lflorea68@yahoo.com (L.F.); vlad.cristiana@gmail.com (C.V.); tanasa_ana_umf@yahoo.com (A.T.); mihai.s.constantin@umfiasi.ro (M.C.); daniel.cioloca@umfiasi.ro (D.C.); codruta.badescu@gmail.com (M.C.B.); feleamag@yahoo.com (M.F.); 2 “Prof. George I.M. Georgescu” Institute of Cardiovascular Diseases Iași, 700503 Iasi, Romania; aminbazyani@gmail.com

**Keywords:** CABG, cardiac rehabilitation, Q10 coenzyme, H-FABP, HAM-D scale, HAM-A scale

## Abstract

(1) Background: The heart-type fatty acid-binding protein (H-FABP) is a specific myocardial biomarker and high levels indicate ischemia regardless of patient-reported symptoms. Concurrently, major adverse cardiovascular events and surgery such as coronary artery by-pass grafting (CABG) cause substantial psycho-emotional distress e.g., depression and anxiety. Comprehensive cardiac rehabilitation is, therefore, essential to both physical and psychological recovery. (2) Methods: This is a unicentric, prospective study on 120 consecutive post-CABG patients undergoing a 6-month cardiac rehabilitation program based on physical exercise, Mediterranean diet principles, and Q10 coenzyme antioxidant supplements. H-FABP levels, depression, and anxiety scores (Hamilton HAM-D and HAM-A scales) were monitored after surgery and at 6 months. (3) Results: Mean H-FABP dropped from 60.56 to 4.81. Physical ability increased from 1–2 to 4–5 METS. Mean depression and anxiety improved from 15.88 to 6.96 and from 25.13 to 15.68, respectively. Median scores went down 50% for depression and 9% for anxiety. Explored associations between H-FABP and psycho-emotional status were statistically insignificant. (4) Conclusions: patients adhered to the program and improved significantly in all studied aspects. Clinical significance is discussed in the context of countries like Romania, where such programs are limited by systemic and financial constraints. Further research directions are identified.

## 1. Introduction

For over 50 years, cardiovascular diseases have been the leading cause of death among all active (adult) population age groups in the U.S. and Europe. Coronary artery disease and acute myocardial infarction occupy the first position in the etiological classification of cardiovascular disability and mortality. Globally, it is estimated that fifty million people suffer from coronary heart disease, and one in four is likely to experience a serious event in the ten years following their diagnosis.

In Romania, over half of deaths were caused by cardiovascular diseases, which places Romania among the countries with populations at high risk of cardiovascular diseases according to the 2020 Guidelines of the European Society of Cardiology (SEC) [1,2,3,4]. In large urban centers such as the city of Iași (the 2nd most populous after the capital Bucharest), life-saving heart surgery and important related healthcare services are readily available. However, the standardized provision of integrated and comprehensive cardiac rehabilitation programs is limited by insufficient funding and systemic inadequacies. From the perspective of a patient recovering from a major cardiovascular event and surgical intervention, access to longer-term support can involve logistical, bureaucratic, and financial hurdles.

### 1.1. The Heart-Type Fatty Acid-Binding Protein (H-FABP) as an Early Indicator of Myocardial Injury

The heart-type fatty acid-binding protein (H-FABP), as with other proteins from the same family, plays a role in the transport of free fatty acid molecules in the cytosol. It is also responsible for the synthesis of membranes and mediators, it influences gene expression, and it protects cells during ischemia by inactivating radicals. It is excreted renally and has a half-life of 20 min [5].

H-FABP levels are known to increase in response to myocardial ischemia, skeletal muscle damage, and vascular damage. A detectable level is reached in the initial period of myocardial necrosis, which means that H-FABP is a sensitive and reliable biomarker that can be used for the early detection of myocardial injury. In patients with perioperative myocardial infarction (PMI), the level of H-FABP is high and it can be used as an earlier telltale sign than the cTnI biomarker to differentiate between patients with and without perioperative myocardial infarction one hour after CABG, compared to increased cTnI levels after about 12 h [6].

Research into the sensitivity of H-FABP also shows that the higher its level during the initial acute event resolved through surgery, the higher the risk of major cardiovascular complications, re-infarction or of death within the following year [7]. In another study, patients with H-FABP levels > 6.48 μg/L were found to be at significantly greater risk of subsequent adverse events [8].

### 1.2. Depression and Anxiety in Cardiovascular Disease

The insidious relationship between cardiovascular disease and altered mood is mediated by inflammation and oxidative stress that together participate in a vicious cycle burdening the CAD patient’s recovery and mental health [9,10,11]. It is, by now, well known that depression is common among patients surviving major cardiovascular events and recovering from surgical interventions such as coronary artery bypass grafting (CABG). Ongoing chest pain, poor functionality, frequent hospitalization, and the high risk of death go hand in hand with deteriorating mental status and quality of life [12]. 

At the same time, research shows that the benefits of anti-depressive medication can reach beyond mood when administered to cardiac patients suffering from depression. For instance, drugs such as sertraline can also improve inflammatory status and endothelial function [13]. Although the physiological connections between an optimistic outlook and cardiovascular outcomes are yet to be fully understood, objective positive biological effects seem to help patients adhere to their treatment regimens, manage stress, and find motivation to incorporate healthy lifestyle changes [14,15].

Two survey tools that can help assess the psycho-emotional outlook of recovering patients are the Hamilton Rating Scale for Depression and the Hamilton Rating Scale for Anxiety, which we shall refer to as HAM-D and HAM-A, respectively [16,17]. First published by Max Hamilton in 1960 as a 17-item questionnaire and subsequently updated on several occasions, HAM-D is used by clinicians to invite adult patients to rate aspects such as mood, feelings of guilt, suicidal ideas, insomnia, agitation or retardation, anxiety, fears, weight loss, somatic symptoms, etc. on a scale of 3 or 5 points, depending on category. The interview takes about 20 min and the total score points to the presence and severity of depression: normal state (0–7), mild (8–13), moderate (14–18), severe (19–22), or very severe (≥ 23) depression. Initially considered the “gold standard” for evaluating depression in clinical research, it has since been criticized as a testing questionnaire in clinical practice for how it emphasizes certain aspects to the detriment of others [12]. Nonetheless, post-CABG patients who entertained an optimistic outlook scored lower on the HAM-D scale, responded better to treatment, and required less frequent hospitalization [18].

The other questionnaire, HAM-A, is used to assess the severity of a patient’s anxiety. It was originally published by Max Hamilton in 1959. Although it is one of the oldest anxiety assessment scales and more such tools have since been developed, HAM-A remains widely used for clinical and scientific purposes. The scale consists of 14 items featuring groups of psychological and somatic symptoms related to experienced agitation and stress. Each group of items is evaluated on a scale from 0 to 4. The total score indicates mild (≤ 17), mild to moderate (18–24), or moderate to severe (25–30) anxiety [17].

### 1.3. The Benefits and Safety of Physical Exercise

Post-CABG rehabilitation by means of physical exercise is considered essential, as it significantly improves the metabolism of the skeletal muscles, stimulates blood flow and capillary density, and facilitates the synthesis and release of nitric oxide to improve angiogenesis and reduce oxidative stress. Physical exertion balances sympathetic and parasympathetic excitation, thus also reducing cardiac arrythmias and ischemia. New guidelines and position papers based on comprehensive reviews of applied research are available to inform clinical practice in their efforts to provide safe, effective physical rehabilitation plans for CAD patients with a history of adverse events and surgery [19,20].

As research has shown, working out is safe with regard to central hemodynamics, left ventricular remodeling, systolic pressure, diastolic function, and myocardial metabolism. Safety has also been demonstrated in the context of patients exercising intensively in real-life settings [21]. In circumstances demanding social isolation, such as the recent Covid-19 pandemic, telerehabilitation can help patients adhere to recommended exercising plans and continue their recovery from home [22].

### 1.4. The Benefits of Adjuvant Antioxidant Treatments

Additionally, antioxidant therapy in patients recovering from major cardiovascular events and life-saving heart surgery promises to contribute positively to their physical rehabilitation and psycho-emotional wellbeing. The benefits of vitamins B, C, and E, as well as coenzyme Q10 (CoQ10), alpha lipoic acid, vitamin A, and zinc are well documented: these supplements enhance each other’s antioxidant properties and protect the body from oxidative stress before and after surgery. Antioxidant therapy is also effective in reducing glutathione levels while boosting catalase after coronary artery by-pass, percutaneous angioplasty, or acute myocardial infarction [23,24].

About the Q10 coenzyme specifically, experts have yet to establish the ideal posology. In clinical trials so far, adults were administered between 50 to 1200 milligrams in one or several doses. A dose of 100–200 milligrams is considered standard. Taking CoQ10 while consuming healthy fatty meals, such as those containing avocado, coconut oil, or olive oil, optimizes its absorption, given that it is soluble in lipids.

Studies into the effects of CoQ10 supplementation have reported significant anti-inflammatory benefits when used as adjuvant therapy prior to heart surgery [25,26]. Also, the monocyte-T-lymphocyte, cytokine or inflammatory hypothesis of depression was confirmed as researchers were able to demonstrate the relationship between inflammatory and oxidative stress, on one hand and depressive mental states on the other [27]. Moreover, plasma CoQ10 deficiency has been investigated and discussed as a factor in pathways linking inflammation and depression, including in cases of treatment resistance [28].

At the same time, low levels of plasma CoQ10 have been found to promote heart failure, while adequate supplementation can help improve systolic function, left ventricular ejection fraction, etc., and prevent coronary artery disease [29]. The neuroprotective properties of this enzyme are by now widely acknowledged, as well, with therapeutic applications in the context of treating chronic fatigue, intolerance to exercise, and various neurodegenerative conditions [30,31].

### 1.5. Study Rationale and Objectives

This is a unicentric, prospective assessment of cardiac rehabilitation and self-reported patient outcomes following CABG surgery and a 6-month program of physical exercise, dietary guidance, and adjuvant antioxidant therapy with Q10 coenzyme supplements.

One primary endpoint was to measure the blood levels of the H-FABP protein as an indicator of acute ischemic status, and then to reassess at a later time well into the recovery period given the marker’s reported predictive value for risk of re-infarction. Concurrently, we aimed to survey the patients’ perceived psycho-emotional status over the same period of time (depression and anxiety scores). The expectation was that all these aspects would improve considerably for patients adhering to the program; exploring to what extent these beneficial effects might be correlated was a secondary endpoint afforded by the twofold study approach.

Knowing also that poor psycho-emotional status (depression and anxiety) has been significantly correlated with pro-inflammatory status, and that both contribute to cardiovascular risk, we were interested in exploring more indirect potential association between H-FABP levels and HAM-D/HAM-A scores. A wider range of biomarkers (pro-inflammatory cytokines or glutathione for oxidative stress) was not available to us due to limited resources, and H-FABP was more novel and intriguing to us at the time.

To the best of our knowledge, this study contributes new data from a geographical area scarcely described and discussed in the literature, (NE) Romania. Moreover, the healthcare resources featured in the study are illustrative of areas where life-saving heart surgery is available, but rehabilitation services are limited and/or separately provided, so cost-effective optimizations, patient adherence, and subsequent benefits are important considerations.

## 2. Materials and Methods

### 2.1. Patient Enrolment, Surgical Treatment and Clinical Assessment

The study was conducted between 01.05.2015–01.03.2017 on consecutive consenting patients who had undergone CABG surgery at the “Prof. Dr. George I.M. Georgescu” Institute of Cardiovascular Diseases in Iași, Romania, within a week of enrolment in the study. The surgical intervention and initial recovery period were followed by in-hospital and then ambulatory cardiac rehabilitation at the Cardiovascular Recovery Clinic of the Clinical Recovery Hospital in Iași.

Other inclusion for criteria were age > 40, body mass index BMI > 25 kg/m^2^, sufficient general fitness to be able to proceed with physical rehabilitation safely, and intellectual aptness to understand the invitation and provide informed consent in writing. The study was approved by the Ethics Committee of the “Grigore T. Popa” University of Medicine and Pharmacy Iași, and it was carried out according to the provisions of the Declaration of Helsinki regarding biomedical research on human subjects.

The exclusion criteria were:

- lack of consent;
- missing data;
- psychiatric conditions;
- physical disabilities justifying the contraindication of physical exercise;
- neoplasms;
- stage 5 chronic kidney disease requiring dialysis.

The CABG surgical technique had been decided by cardiac surgeons starting from the patient’s profile and symptoms on admission, which prompted routine and specialized investigations. Age, sex, and history of chronic conditions known to increase cardiovascular and psycho-emotional risk were considered: elevated blood pressure (> 140/85 mmHg), diabetes mellitus, metabolic syndrome, chronic kidney disease, according to international definitions [32,33,34].

The transthoracic echocardiography included the visual inspection of 2D and continuous Doppler imaging to assess heart structures and motion, such as ventricle size, aortic valve function, etc., as well as to establish the presence of heart failure according to the guidelines of the New York Heart Association (NYHA) [35]. Peripheral arterial disease was diagnosed by angiography. Laboratory tests included routine hematological and biochemical parameters (lipid profile, coagulation, inflammation, renal and hepatic function).

### 2.2. Surgical Intervention

The interventions consisted of on-pump CABG surgery via aorto-atrial cannulation and systemic hypothermia. The pump flows were fixed to maintain a heart rate of more than 2.4 L/min/m^2^. Myocardial protection was achieved by intermittent administration of the antegrade degree of a hypothermic cardioplegic solution, St. Thomas II. The pump circuit was primed with Ringer’s solution, heparin, and mannitol. Any artery with an obstruction of more than 80% in coronary angiography was grafted. Complete revascularization was provided in all patients, using the left internal mammary artery, the right internal mammary artery (Y grafts) and the radial artery, respectively.

### 2.3. Postoperative Treatment and Cardiac Rehabilitation

After surgery, the patients were monitored echocardiographically and enrolled in a cardiac rehabilitation program. The administered medication included antiplatelet agents (e.g., acetylsalicylic acid 75 mg/day), statins (e.g., atorvastatin 20 mg/day), beta blockers (e.g., bisoprolol 5 mg/day), sartans (e.g., candesartan 8 mg/day), and diuretics (e.g., 50 mg spironolactone and 20 mg furosemide per day). Medication regimens were individualized. For example, patients with chronic kidney conditions were not given sartans and diuretics. Also, calcium channel blockers were an alternative to beta blockers to manage blood pressure and avoid bronchospastic manifestations in patients with pulmonary conditions and allergies.

The cardiac rehabilitation program consisted of three subsequent phases summarized in Table 1 and was implemented in collaboration with the local clinical recovery hospital. In the first phase post-surgery, including the time spent in intensive care, the patients were guided through breathing exercises and low intensity kinesiotherapy (passive and active). As the patients improved, they were transferred to the clinical recovery hospital and then, upon further recovery, were discharged home. Throughout phases II and III, the patients attended regular kinesiotherapy sessions at the partner hospital. They were also instructed and encouraged to do more exercises at home, and members of the medical team typically kept in contact by phone.

The patients’ progress in terms of physical and cardiac fitness was assessed by medical and kinesiotherapy staff based on the patients’ monitored performance of tasks such as standing, walking, squatting, bicycling, jogging, etc. Relative to the resting rate of sitting in bed (1 metabolic equivalent, or MET), standing up and light walking corresponded to 2 METs, while walking and bicycling at a moderate pace were rated as 4–5 METs, depending on intensity.

Regarding the patients’ nutrition, the hospital’s dietician recommended variations of the Mediterranean diet, depending on each patient’s status, pathology, and personal perspective. The diet plans focused on reducing the intake of lipids and salt, which are cultural staples in Romanian cuisine. Also, the patients were explained the benefits of the Q10 coenzyme as a supplement and were prescribed 200 mg daily for a period of 6 months.

Anti-depressive medication was taken into consideration if a patient’s initial mood would not improve spontaneously with cardiac rehabilitation and overall progress. Similarly, benzodiazepines with mild sedative effects, such as alprazolam, were an option to facilitate relaxation, reduce anxiety, or help with sleeplessness, if necessary.

### 2.4. H-FABP Data Collection

The blood samples used to measure H-FABP were drawn within the first 6 h after the intervention, while the patients were in ICU (phase I). Another set of samples was collected six months later (phase III). All these were frozen immediately at 20 °C (the required temperature for storage periods longer than 24 h), because the necessary test kits became available a year later. The samples were processed with Randox kits according to the manufacturer’s instructions, using the latex-enhanced immunoturbidimetric assay method of running standard reagents on clinical chemistry analyzers. More frequent sampling was unavailable due to financial limitations (see below).

### 2.5. Psychological Assessment and Data Collection

The HAM-D and HAM-A questionnaires were used to survey the patients after their surgical interventions (phase I) and then again during their rehabilitation program supplemented with antioxidant therapy (phase III). The scores were computed as can currently be seen at https://www.mdcalc.com/hamilton-depression-rating-scale-ham-d and https://psychology-tools.com/hamilton-anxiety-rating-scale (both links were last accessed on 1 June 2022, to check the availability of the information) [16,17].

### 2.6. Statistical Analysis

The database was initially compiled in Microsoft Office Excel version 2010, and the statistical analysis was done in IBM SPSS Statistics for Windows, version 20 (IBM Corp., Armonk, NY, USA). The averages, frequencies, standard deviations, differences between maximum and minimum values, etc., were calculated, and the results were compared between different clinical phases of patient recovery. Multivariate linear regression analysis was performed to evaluate correlations between H-FABP levels and psycho-emotional risk scores in patients receiving antioxidant treatment. Two independent operators performed the analysis and any noteworthy differences prompted verifications and recalculations by a third.

## 3. Results

### 3.1. General and Clinical Characteristics Post CABG Surgery (Enrolment—Phase I)

In all, 159 patients were consecutively treated and screened as explained during the studied period. Of them, 120 patients met the inclusion criteria and provided complete data. A total of 19 patients either did not consent or withdrew in the first phase after surgery. In both types of cases, the patients preferred to be discharged directly home rather than be transferred to the Clinical Recovery Hospital to continue their recovery according to how public healthcare services were organized at the time (this transfer could not be avoided).

The 120 consenting patients who stayed with the program were 65 men and 67 women between 40 and 80 years old (their mean age was 65.93 ± 9.83). The mean body mass index (BMI) was 29.4 ± 3.02 kg/m^2^. Slightly more than half of the patients (63) were diabetic, and the mean blood sugar level was 138.53 ± 57.43 mg/dL. The mean total cholesterol was 181.54 ± 46.33 mg/dL. Metabolic syndrome was common based on a combination of elevated triglycerides, fasting plasma glucose, and blood pressure levels in addition to central obesity. Also, 73 patients (60.3%) were smokers. Except in two cases, all the patients were hypertensive (> 140/85 mmHg) and 89.8% presented moderate left ventricular concentric hypertrophy (mean left ventricular mass index value of 143.31 ± 46.40 g/m^2^). Also, a majority of patients had atherosclerotic disease; notably, 76 patients (62.8%) had a history of myocardial infarction, 6 patients (5%) had suffered a stroke, and three had peripheral arterial disease. As many as 81 patients were admitted with NYHA class ≥ II (67%) and had a mean left ventricular ejection fraction (LVEF) of 43.47 ± 7.32%.

Table 2 and Table 3 below provide a comparative view of biochemical, and echocardiographic characteristics of the study group upon enrolment after surgery (phase I) and again in phase III.

### 3.2. Evidence of Cardiac Rehabilitation and Other Clinical Outcomes

In the six months of cardiovascular rehabilitation program post CABG surgery, none of the patients were readmitted with any major adverse cardiac events. Also, none presented with or reported signs and symptoms that would suggest an aggravation or complication of their condition. Dropping out did not occur in phases II and III. In other words, once the patients overcome the initial hurdles of the surgery and transfer, their adherence to the program was excellent.

57 out of 66 patients with atrial fibrillation (86.36%) converted to sinus rhythm, and 7 echocardiographic parameters underwent substantial changes. The left ventricular end-diastolic and end-systolic diameters (LVEDD and LVSDD), the thicknesses of the interventricular septum (IVS) and of the posterior wall (PW) of the left ventricle, the left ventricular mass and mass index (LVMI) decreased in phase III, while the ejection fraction (EF) and fractional shortening (FS) increased. The statistical significance of the results summarized in Table 2 below suggests that these changes were not random but outcomes of the rehabilitation program and normal healing processes.

In addition, almost all the patients’ blood test results improved significantly in phase III compared to phase I, except for the levels of gamma-glutamyl transferase (GGT), which remained the same. Blood urea decreased significantly, and the renal filtration function was significantly better. The lipid profile was also significantly ameliorated, although patients whose initial levels were above normal did not fall back within the cardiovascular risk-free range. These results are summarized in Table 3.

Regarding physical activity, a week after the myocardial infarction and subsequent intervention (phase I), the patients could only handle minimal effort corresponding to the metabolic equivalent of sitting or light walking (1 or 2 METs). In phase III, all the patients who had been in this situation were able to perform activities requiring substantially more effort (4 or 5 METs). More than 50% of patients could manage 5 METs six months after their CABG surgery.

### 3.3. H-FABP Levels in Phase I and Phase III

**Figure 1:**
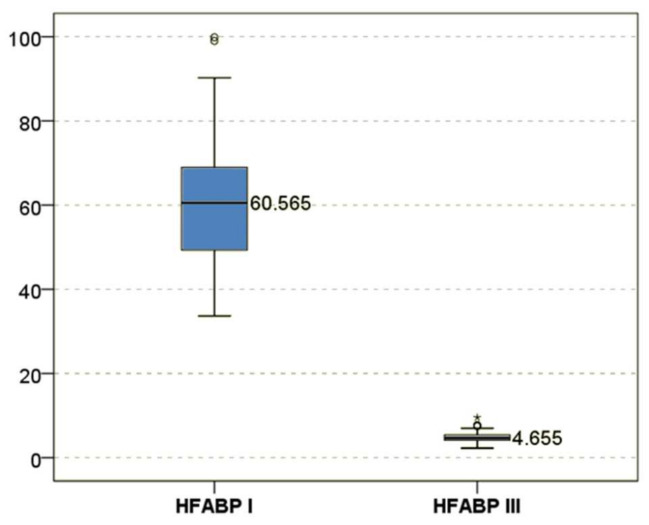
H-FABP differences between the two studied phases (*N* = 120).

The samples collected in phase I revealed a mean level of H-FABP soon after surgery of 68.39 units (median value 60.56), while in phase III the mean level dropped substantially to 4.81 units (median value 4.65) (Figure 1).

### 3.4. Depression and Anxiety Scores

Before describing the results of self-reported psycho-emotional status, it is worth noting that the patients did not manifest a need or explicit interest in antidepressant or anxiolytic medication. The only exception was in phase I, when patients struggling to fall sleep were given alprazolam in the form of 0.25 mg Xanax pills. This was phased out as soon as possible to prevent the onset of addictive effects, and the patients were advised against using such sedatives without first consulting with their physician. According to our knowledge, by phase 3, all the patients relied exclusively on natural remedies for sleep-inducing relaxation, if necessary.

**Figure 2:**
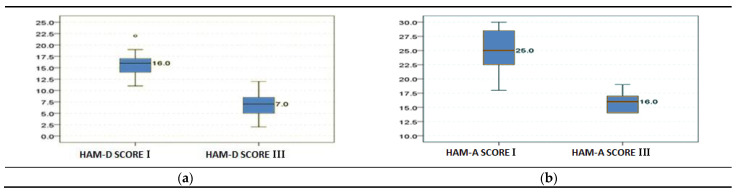
Differences in (**a**) depression and (**b**) anxiety scores between the two studied phases (*N* = 120).

In the first week post-CABG (phase I), the patients’ responses to the HAM-D questionnaire resulted in scores ranging from 11 to 22 points (mean value 15.88 ± 2.189), which indicates that the depression experienced by 50% of the patients was mild to severe. After 6 months of cardiac recovery, when the HAM-D questionnaire was applied again, the scores ranged from 2 to 12 only (mean value 6.96 ± 2.696), suggesting substantial improvement towards normal mood or mild depression among 50% of the group. A significant reduction by 56.17% of the median HAM-D values (*p* < 0.05) is another noteworthy evidence of improvement from phase I to phase III (Table 4 and Figure 2a). 

Regarding the patients’ experience of anxiety as rated using the HAM-A questionnaire, in the first phase of the cardiac rehabilitation program the scores ranged from 18 to 30 (mean value 25.13 ± 3.541), which was indicative of mild-to-moderate and moderate-to-severe anxiety. In phase III, the patients scored between 14–19 (mean value 15.68 ± 1.442), which was a notable improvement towards milder anxiety alongside the aforementioned amelioration of depression, even if the 9% decrease of the median HAM-A value in phase III was not as statistically relevant (Table 4 and Figure 2b).

For both depression and anxiety, we analyzed the differences between phases in each case using the Wilcoxon Signed Ranks Test. The fact that ties (cases of equal values) were absent suggests that the psychological status of every patient evolved favorably (see Table 5).

Also, the Wilcoxon W+ test revealed that the distributions of scores were similar in both phases for both depression and anxiety, which further confirms our observation that the psychological benefits were coherent and consistent across the group and studied variables (see Table 6).

### 3.5. Analysis of H-FABP Levels in Relation to Other Data Sets, including Psycho-Emotional Status

Using linear regression and univariate generalized linear models, we assessed multiple variables for their clinical prediction of myocardial injury expressed through H-FABP levels. The results are summarized in Table 7, with statistically significant values highlighted in bold.

These *p* values confirm yet again the well-established fact that diabetes and hypertension, for instance, are risk factors for myocardial injury. As such, they provide a reference point in our assessment of other potential associations, specifically with the patients’ psycho-emotional status at different stages of recovery.

Regarding the patients’ H-FABP levels and their depression scores soon after their CABG intervention (phase I), our analysis revealed a negative association of −0.00186 (HAM-D) and a statistically insignificant F value of 0.98 (> 0.05), which does not point to any noteworthy correlation between these two variables. The results were similar for the association between H-FABP and HAM-A scores in the initial phase of cardiac rehabilitation and antioxidant treatment; the value of the correlation was −0.06441, F value 0.484617 (> 0.05).

**Figure 3:**
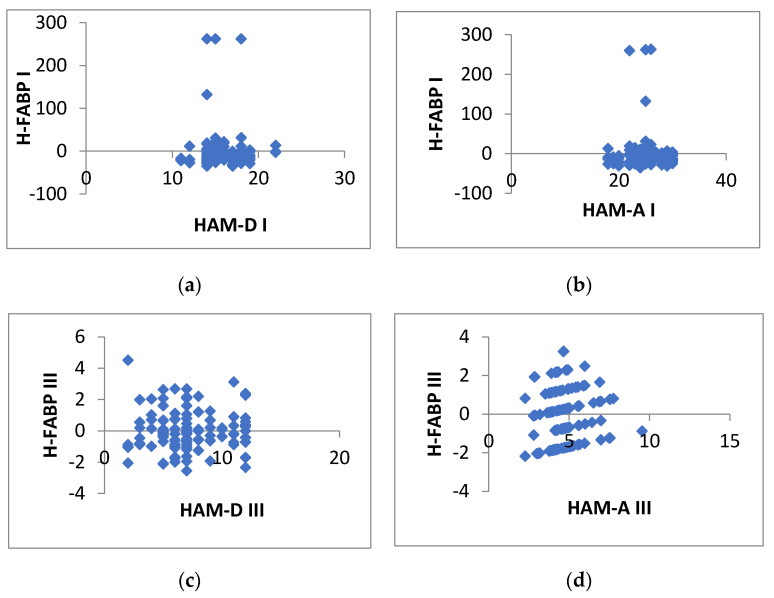
Insignificant negative associations between H-FABP levels and psycho-emotional scores: (**a**) H-FABP and HAM-D in phase I; (**b**) H-FABP and HAM-A in phase I; (**c**) H-FABP and HAM-D in phase III; (**d**) H-FABP and HAM-A in phase III (*N* = 120).

After six months of cardiac rehabilitation and antioxidant treatment, the same kind of associations were still negative and statistically insignificant. The correlation between patients’ H-FABP levels and their depressions scores was −0.15354 (HAM-A) with an F value of 0.094079, while for H-FABP and HAM-D it was −0.09536 with F 0.30016 (> 0.05). The plotted results can be seen in the set of figures below (Figure 3a–d).

## 4. Discussion

This was a single-center, prospective study with a patient follow-up period up to 6 months, aiming to assess the myocardial injury/recovery marker H-FABP and the psycho-emotional status of patients after life-saving CABG surgery, as they adhered to a cardiac rehabilitation program of kinesiotherapy, dietary advice, and antioxidant therapy with the Q10 coenzyme. 

The program built on a standard provision of cardiac rehabilitation (limited in scope and duration to the initial post-surgery phase) by extending it in collaboration with the clinical recovery hospital from the same city. It was further enhanced by supporting patients towards home exercises and healthier eating habits. Also, the decision to prescribe additional antioxidant therapy was to promote physical and psychological recovery in a way that would be easy for our patients to comply with, informed by the reported benefits of this enzyme such as referenced earlier.

In the first phase after the intervention, H-FABP levels were elevated in all cases. Inflammation, oxidative stress, and apoptosis can increase the permeability of cardiac cells, thus contributing to the release of H-FABP [18]. Also, depression and anxiety were more intense and widespread in the early days following the intervention. By the end of the monitored period, objectively measured H-FABP values had substantially decreased, concurrent with significant improvements in self-reported depression and anxiety scores, leading us to hypothesize a potentially useful association between the two. However, inferential statistical analysis did not necessarily confirm this. Correlation values of interest from this point of view were not significant, while others helped anchor the output data (e.g., significant associations with diabetes and hypertension, as expected).

Our patients were advised to present immediately in case of pain or other aggravating symptoms that would alert us to a potential graft failure or re-infarction: there were no such cases and no deaths. To be able to ascertain if the patients suffered any silent ischemic manifestations, a shortly persisting biomarker such as H-FABP would have to be sampled repeatedly, such as when patients present for follow up and/or kinesiotherapy sessions. Detecting elevated H-FABP in an unassuming, asymptomatic patient would alert the clinician to the possibility and risks of another adverse event. This would give the medical team the opportunity to intervene immediately, but our experience is that securing the necessary funding for such testing capabilities can be difficult and unpredictable.

Importantly, cardiovascular medicine is beginning to express increasing concern for the psycho-emotional challenges associated with coronary artery disease, a trend to which we subscribe fully. The patients’ survival, physical and social rehabilitation, as well as quality of life can be substantially enhanced through the early recognition, diagnosis, and intervention related to depression and anxiety. Consensus is growing among physicians that former practices of treating the disease and not the individual should become a thing of the past.

In patients who undergo CABG surgery, depression and anxiety have been identified as independent risk factors for hospital readmission, morbidity, and cardiac mortality. These can affect up to 40% of CABG patients and are also correlated with a decrease in quality-of-life scores and health care costs per capita. Research linking pre-surgical depression with longer hospitalization times after CABG interventions can be built upon and expanded, then applied towards more holistic diagnostic, treatment, and follow up approaches [36,37,38].

In our study, we found that the patients’ moods improved noticeably as they recovered from surgery and continued to make progress after discharge. Beyond a few cases of using a sedative to facilitate sleep, none of the patients enrolled in our study showed need or explicit interest in taking antidepressant or anxiolytic medication. This result should be seen in the wider context of Romania and other countries, where depression is still a cultural taboo and source of social stigma preventing many people from acknowledging their mental health issues and seeking specialized help. Therefore, we cannot claim that our survey data is truly and entirely reflective of the patients’ internal emotional realities. On the other hand, the results of our study point to the underlying psychological benefits of cardiac rehabilitation programs based on physical exercise, healthy eating, antioxidant therapy. To our knowledge, this is the first study focusing on possible associations between H-FABP levels and psycho-emotional status in CABG patients who, in addition to enrolling in a standard cardiac rehabilitation program, agreed take CoQ10 supplements daily as adjuvant antioxidative therapy. Upon comparing the phase I and phase III results, we noticed a significant reduction of close to 50% in median depression scores and of almost 9% in median anxiety scores. We believe this reflects the physical and psychological benefits of a recovery program that builds on limited standard provision through inter-hospital collaboration, reasonable dietary advice, and antioxidative treatment supplementation. Certain aspects can be further improved without mobilizing additional resources. For instance, future eating and nutrition advice can build on known preferences and habits (e.g., religious fasting days and periods) to promote the principles of plant-based diets and restricted mealtimes, whose benefits are now better known [39].

Enrollment in cardiac rehabilitation programs in some countries is yet to reach optimal levels despite scientifically demonstrated benefits and clinicians’ recommendations [19,20,21,40]. Our findings can help promote more effective communication featuring psychological benefits as a motivating and mobilizing factor for reluctant patients. This is even more important in the recent context of the COVID-19 pandemic, when quarantine and social isolation measures have limited access to healthcare and provided fertile ground for depression, anxiety, and other mental health issues.

This study is subject to several limitations which provide additional context for interested readers to better understand the methodological choices made and interpret the results. Due to the complex, multidisciplinary nature of the cardiac intervention and recovery program, a sample size of 120 patients was collectively agreed to be a large enough to achieve statistical significance, yet reasonably small to afford the labor and costs of data collection, laboratory testing, patient monitoring (including by phone), etc. Also, regarding the adjuvant antioxidant therapy, the uniform administration of Q10 enzyme supplements to all the patients was a decision focused on clinical benefits yet lacking the scientific foresight that would have resulted in organizing a control and/or placebo group.

Moreover, financial constraints, as well as unforeseen delays and opportunities in the purchase of assay kits caused the team to have to adapt and refocus initial research interests. This explains why there was no sampling for H-FAPB levels on admission (before CABG surgery) or more often during the studied period. The same is true for why other relevant biomarkers were not measured, such as levels of glutathione and malonyl dialdehyde as indicators of oxidative stress, or levels of cytokines for assessing pro-inflammatory status.

Even if these implementation challenges invite a more speculative discussion than intended, the results are clinically encouraging and scientifically interesting to justify communication and to inform further research. All in all, from a methodological standpoint, the observational and prospective nature of this study limits its scope to hypothesizing about potentially meaningful associations between otherwise clearly demonstrated substantial benefits. To investigate them further, it is advisable to plan and secure in advance the necessary budgets and resources for all the aspects and stages of such research.

## 5. Conclusions

H-FABP is a direct marker of cardiac injury. In our study, it was used to also re-evaluate cardiac performance in CABG patients undergoing cardiac rehabilitation treatment. Beyond the initial event prompting life-saving investigations and surgical intervention, continuing to measure H-FABP levels during the rehabilitation period can be informative even if patients do not report symptoms, to confirm myocardial recovery and to screen for silent ischemic manifestations. At the same time, effective cardiovascular rehabilitation plays a key role in improving the psycho-emotional status of CABG patients. This can be reliably assessed with the widely used Hamilton scales for depression and anxiety, HAM-D and HAM-A, respectively.

Our study shows that cardiac rehabilitation based on kinesiotherapy, dietary advice, and adjuvant antioxidant therapy improves myocardial health and psycho-emotional status over time. While such results are not new, the integrated analysis of myocardial injury and psycho-emotional status provides interesting directions for further research, e.g., trialing coQ10 supplementation with a control/placebo group, measuring H-FABP levels more frequently during rehabilitation, including oxidative stress and inflammation markers in similar analyses of physical and psycho-emotional status, etc. Hypothesized associations between myocardial injury and psycho-emotional status were not substantiated on this occasion, although the data confirmed well-known relationships. Also, effects could not be isolated and measured due to methodological and financial limitations that should be considered when organizing future research.

Last but not least, the study contributes data from Romania, where our program built on a minimal standard provision of cardiac physical rehabilitation in a context of systemic limitations that are not unique to our country. Using available resources and collaboration opportunities, the patients were more substantially supported through gradual, guided recovery and manageable lifestyle changes. More broadly, the results also reinforce the importance of ongoing psychological assessment in the clinical management of patients recovering from heart surgery.

## Figures and Tables

**Table 1:** Phases of the cardiac rehabilitation program and additional antioxidant treatment.

Description	Phase I	Phase II	Phase III
Duration	weeks 0–2	weeks 3–10/12	week 12–month 6
Location	first 3–5 days in ICU, then 7–10 days post-ICU	clinical recovery center and ambulatory care	in hospital, individually or in groups
Objectives	prevent the effects of deconditioning and immobility	restore previous ability level	attain and maintain best performance, achieve tertiary prevention
Methods	kinesiotherapy, walking, respiratory gymnastics	physical exercise, walking, swimming, etc.	physical exercise, walking, swimming, etc.
Antioxidant treatment	Q10 coenzyme 200 mg/day	Q10 coenzyme 200 mg/day	Q10 coenzyme 200 mg/day

**Table 2:** Echocardiographic results in the two studied phases (*N* = 120).

Echocardiographic Data	Phase	Value	St. Dev.	Std. Error	*p* Value
LVEDd (mm)	phase I	52.14	8.914	0.891	0.000
	phase III	48.89	9.195	0.919	
LVESDd (mm)	phase I	36.65	8.776	0.878	0.000
	phase III	33.02	9.497	0.950	
IVS (mm)	phase I	13.10	2.389	0.239	0.000
	phase III	12.26	2.623	0.262	
PW (mm)	phase I	12.25	1.445	0.145	0.000
	phase III	11.49	1.648	0.165	
EF (%)	phase I	43.55	7.615	0.761	0.000
	phase III	52.35	10.593	1.059	
FS (%)	phase I	25.55	6.420	0.642	0.000
	phase III	27.78	6.907	0.691	
LVM (g)	phase I	276.43	94.761	9.476	0.000
	phase III	228.86	89.106	8.911	
LVMI *(*g/m^2^)	phase I	143.11	47.403	4.740	0.000
	phase III	120.45	46.232	4.623	

**Table 3:** Biochemical results in the two studied phases (*N* = 120).

Biochemical Data	Phase	Value	95% Lower	CI Upper	Median	Std. Dev.	Min	Max
ALT (U/L)	phase I	39.74	33.02	46.46	30.00	37.16	15.00	285.00
	phase III	26.11	23.48	28.73	22.50	14.52	9.00	93.00
AST (U/L)	phase I	34.63	29.89	39.38	27.50	26.25	17.00	164.00
	phase III	24.53	21.41	27.66	20.00	17.30	10.00	159.00
GGT (U/L)	phase I	40.93	36.53	45.32	33.50	24.32	15.00	180.00
	phase III	39.23	31.21	47.25	30.00	44.37	12.00	456.00
Total bilirubin (mg/dL)	phase I	0.74	0.70	0.78	0.79	0.21	0.35	1.07
	phase III	0.59	0.55	0.62	0.55	0.18	0.31	1.04
Direct bilirubin (mg/dL)	phase I	0.37	0.33	0.41	0.30	0.23	0.12	1.00
	phase III	0.22	0.20	0.23	0.18	0.07	0.12	0.42
Total Cholesterol (mg/dL)	phase I	181.54	173.16	189.92	178.50	46.37	104.00	390.00
	phase III	168.10	159.27	176.93	155.00	48.86	102.00	318.00
HDL-cholesterol (mg/dL)	phase I	40.19	36.35	44.04	40.00	21.27	10.00	180.00
	phase III	50.33	45.72	54.94	45.00	25.50	4.04	200.00
LDL-cholesterol (mg/dL)	phase I	144.26	138.71	149.81	150.00	30.71	100.00	250.00
	phase III	122.03	116.81	127.24	110.00	28.85	90.00	234.0
Triglycerides (mg/dL)	phase I	147.95	137.66	158.24	136.00	56.95	69.00	300.00
	phase III	132.64	122.33	142.94	116.00	56.99	43.00	294.00
Urea (mg/dL)	phase I	45.42	42.78	48.05	43.00	14.56	20.00	86.00
	phase III	41.45	39.27	43.63	40.00	12.07	18.00	65.00
Creatinine (mg/dL)	phase I	1.28	1.20	1.36	1.20	0.44	0.80	3.80
	phase III	1.09	1.05	1.12	1.08	0.21	0.60	1.57

**Table 4:** Depression and anxiety scores in the two studied phases (*N* = 120).

Survey Tools	Phase	Mean	Std. Dev.	Min	Max
HAM-D for depression	phase I	15.88	2.189	11	22
	phase III	6.96	2.696	2	12
HAM-A for anxiety	phase I	25.13	3.541	18	30
	phase III	15.68	1.442	14	19

**Table 5:** Wilcoxon S-R test results–ranks table output.

Data Sets	Ranks	*N*	Mean Rank	Sum of Ranks
HAM-D scores phase III–I	Negative ranks	120 ^a^	50.50	5050.0
	Positive ranks	0 ^b^	0.00	0.00
	Ties	0 ^c^		
	Total	120		
HAM-A scores phase I–I–I	Negative ranks	120 ^d^	50.50	5050.00
	Positive ranks	0 ^e^	0.00	0.00
	Ties	0 ^f^		
	Total	120		

^a^. HAM-D scores_phase III < HAM-D scores_phase I; ^b^. HAM-D scores_phase III > HAM-D scores_phase I; ^c^. HAM-D scores_phase III = HAM-D scores_phase I. ^d^. HAM-A scores_phase III < HAM-A scores_phase I; ^e^. HAM-A scores_phase III > HAM-A scores_phase I; ^f^. HAM-A scores_phase III = HAM-A scores_phase I.

**Table 6:** Further statistical analysis of score distributions.

Wilcoxon Test Results	HAM-D Scores Phase III—I	HAM-A Scores Phase III—I
**Z**	**−8.718a**	**−8.702a**
**Asymp. Sig. (2-tailed)**	**0.000**	**0.000**

a—based on positive ranks.

**Table 7:** Predictors for myocardial injury—univariate analysis (*N* = 120).

Assessed Variables and H-FABP Levels	*p* Value
Smoking	0.18
**Myocardial infarction**	**0.05**
**Hypertension**	**0.01**
Left ventricular mass index	0.30
Peripheral artery disease	0.24
Stroke	0.97
**Diabetes mellitus**	**0.01**
**Creatinine clearance**	**0.02**
Left ventricular ejection fraction	0.27
Depression	
HAM-D scores in phase I	0.9
HAM-D scores in phase II	0.09
Anxiety	
HAM-A scores in phase I	0.4
HAM-A in phase II	0.3

## Data Availability

The data presented in this study are available on request from the corresponding author. The data are not publicly available due to the confidential nature of patient medical records.
